# Surgical impact of bilateral transient occlusion of uterine and utero-ovarian arteries during laparoscopic myomectomy

**DOI:** 10.1038/s41598-024-57720-9

**Published:** 2024-03-25

**Authors:** Enrique Moratalla-Bartolomé, Jesús Lázaro-de-la-Fuente, Irene López-Carrasco, Elena Cabezas-López, Jose Carugno, Javier Sancho-Sauco, Irene Pelayo-Delgado

**Affiliations:** 1 Department of Obstetrics and Gynecology, Ramón y Cajal University Hospital, 3428034 Madrid, Spain; 2 Department of Obstetrics and Gynecology, HM Montepríncipe-Sanchinarro University Hospital, 3428050 Madrid, Spain; 3 Minimally Invasive Gynecology Division, Department of Obstetrics, Gynecology and Reproductive Sciences, University of Miami, Florida, USA; 4 Department of Obstetrics and Gynecology, Ramón y Cajal University Hospital, Alcalá de Henares University, 3428034 Madrid, Spain

**Keywords:** Blood loss, Temporary artery ligation, Uterine fibroids, Anatomy, Diseases, Health care, Medical research, Signs and symptoms

## Abstract

The objective of this article is to compare the amount of intraoperative blood loss during laparoscopic myomectomy when performing bilateral transient clamping of the uterine and utero-ovarian arteries versus no intervention. It´s a randomized controlled prospective study carried out in the Department of Obstetrics and Gynecology Ramón y Cajal University Hospital and HM Montepríncipe-Sanchinarro University Hospital, Madrid, Spain, in women with fibroid uterus undergoing laparoscopic myomectomy. Eighty women diagnosed with symptomatic fibroid uterus were randomly assigned to undergo laparoscopic myomectomy without additional intervention (Group A) or temporary clamping of bilateral uterine and utero-ovarian arteries prior to laparoscopic myomectomy (Group B). Estimated blood loss, operating time, length of hospital stay, and postoperative hemoglobin values were compared in both groups. The number of fibroids removed was similar in both groups (*p* = 0.77). Estimated blood loss was lower in the group of patients with prior occlusion of uterine arteries (*p* = 0.025) without increasing operating time (*p* = 0.17) nor length of stay (*p* = 0.17). No patient had either intra or postoperative complications. Only two patients (2.5%) required blood transfusion after surgery. We conclude that temporary clamping of bilateral uterine arteries prior to laparoscopic myomectomy is a safe intervention that reduces blood loss without increasing operative time.

## Introduction

Uterine leiomyomas or fibroids are the most common benign tumors of the uterus, found in more than 70% of middle-aged women^1^. Although many fibroids are asymptomatic, 25–50% of cases can cause abnormal uterine bleeding, pelvic pain and pressure, urinary and intestinal symptoms, and/or infertility problems^1^. The treatment of women with uterine leiomyomas should be individualized and is usually dictated by the patient's age and desire for future fertility, the characteristics of the fibroid (size, number, and location), the clinical symptoms, and the availability of different therapeutic options^2^. Surgical treatment includes hysterectomy as a definitive procedure in women who do not wish to preserve future fertility, while myomectomy can be an option for patients with unfulfilled reproductive desires. Laparoscopic myomectomy versus the open laparotomic approach has demonstrated shorter hospital stays, less blood loss, and fewer needs for blood transfusions^3^. However, intraoperative blood loss is one of the most common complications of laparoscopic myomectomy^4^. Different methods have been proposed for reducing bleeding during surgery, including temporary occlusion of the uterine and utero-ovarian arteries (LOUA). The efficacy and safety of this technique has not been formally investigated.

The aim of this study is to compare the difference in intraoperative blood loss and operative time when performing bilateral temporary clamping of the uterine and utero-ovarian arteries versus no intervention prior to laparoscopic myomectomy.

## Methodology

A prospective longitudinal study of 80 consecutive patients with symptomatic fibroids who were scheduled to undergo laparoscopic myomectomy were randomly assigned to one of two groups. Those assigned to group A underwent laparoscopic myomectomy without any additional intervention. Patients assigned to group B underwent bilateral temporary clamping of the uterine arteries and utero-ovarian ligaments prior to initiate the laparoscopic myomectomy. The study took place in two tertiary referral centers in Madrid, Spain (Ramón y Cajal University Hospital and HM Montepríncipe-Sanchinarro University Hospital) between March 2020 and December 2022.

Written informed consent to participate in this study was obtained from all patients before randomization. Institutional review board approval was obtained from both participating centers (Ramón y Cajal University Hospital: date 24/02/2020 ACTA 382 and HM Montepríncipe-Sanchinarro University Hospital: 20.01.1492-GHM). The study was performed in accordance with relevant guidelines and regulations and with the Declaration of Helsinki. CONSORT reporting guidelines were followed (Schulz KF, Altman DG, Moher D, for the CONSORT Group. CONSORT 2010 Statement: updated guidelines for reporting parallel group randomised trials).

The study was registered in ClinicalTrials.gov (NCT05994560, available at https://classic.clinicaltrials.gov/ct2/show/NCT05994560?cond=Laparoscopic+Myomectomy%2C&cntry=ES&draw=2&rank=1, date of registration 16/08/2023). It was also registered at the ISRCTN registry on 22/11/2023 Protocol/serial no.: 324–19 available at https://www.isrctn.com/ISRCTN89994856

### Inclusion and exclusion criteria

Women included had at least one symptomatic myoma after failing medical treatment and desire for uterus preservation. Patients were excluded if they were deemed not good candidates for laparoscopic myomectomy, and those who desired definitive surgical management with hysterectomy.

Prior to randomization, clinical data was collected, including age, height, weight, race, age of menarche, and consumption of tobacco and alcohol. Transvaginal/transabdominal ultrasound and/or Magnetic Resonance Image (RMI) reported the number of fibroids, their size, and location categorized according to the FIGO fibroid classification system^5^. A preoperative laboratory analysis, including the hemoglobin level, was collected within 24 h before the surgery, and it was repeated on postop day 1. The need for blood transfusion, surgical time, days of hospitalization, and improvement of symptoms were assessed. Blood loss during surgery was calculated by the amount of blood aspirated minus the amount of fluid used for irrigation during laparoscopy.

### Surgical technique

The surgery was performed on all the patients by a surgeon with more than 15 years of experience in the field of gynecologic laparoscopic surgery (EMB).

The pneumoperitoneum was obtained using the Verres needle in the upper left quadrant at the point of Palmer’s point. Once the pneumoperitoneum was developed, we introduced a 12 mm optic trocar in the umbilicus for normal size uteri, while in cases with large myomas, it was placed above the umbilicus to obtain better visualization of the operative field. Three additional ports were placed: a 5 mm trocar in each lower quadrant and a 12 mm suprapubic port. A uterine manipulator was also placed. Methylene blue was placed inside the uterine cavity to facilitate the inspection of the integrity of the uterine cavity at the end of the procedure.

**Figure 1:**
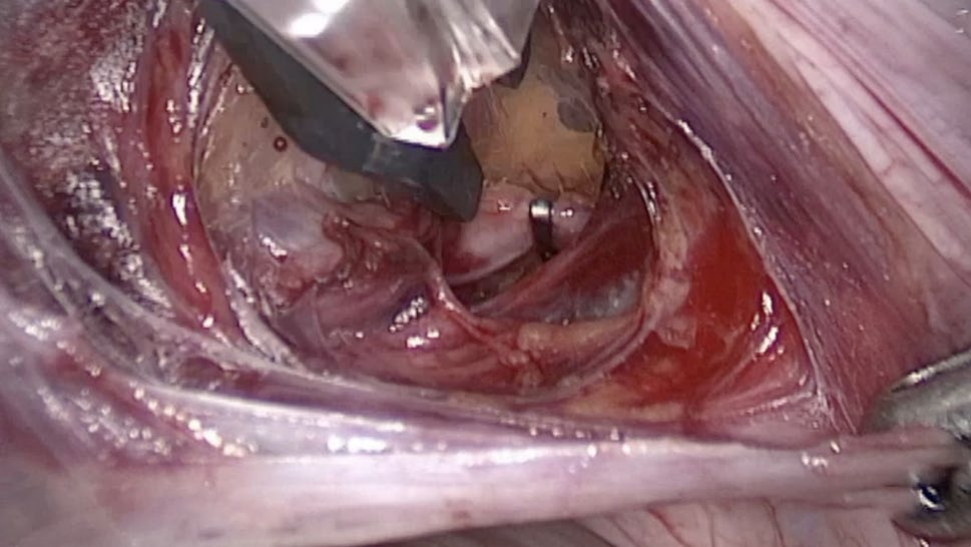
Laparoscopic view of the clip located at the uterine artery.

Laparoscopic temporary occlusion of bilateral uterine arteries was performed following a lateral, posterior, or anterior approach according to the size, number, and location of fibroids and the ability to gain safe access to the uterine artery (Fig. 1).

In the lateral approach, the technique described by Liu et al.^6^ was followed. First it was identified the triangle formed by the external iliac vessels (lateral), the round ligament (anterior), and the fallopian tube (superior) in continuity to the infundibular-pelvic ligament. An incision is made on the pelvic parietal peritoneum identifying the obliterated umbilical ligament. When medially dissected, the uterine artery is visualized, which must be differentiated from the ureter (vermiculation), when identified, the 10 mm Endo Clip™ MedTronic, are placed.

The posterior approach was described by Alborzi et al.^7^. In this case, the ureter, located retroperitoneal in the lateral pelvic wall, is identified. A peritoneal incision is made above the ureter. The ureter is lateralized, and the uterine artery is identified and dissected, at which point the clips are placed.

For the anterior approach, described by Aust et al.^8^, the bladder must be visualized, which is located between the two obliterated umbilical ligaments. An incision is made in the peritoneum above the obliterated umbilical ligament, and the origin of the uterine artery is traced up to its origin. The ureter, when using this approach, is found medially and inferior to the incision.

The laparoscopic myomectomy was performed by making a longitudinal incision on the uterine serosa above the fibroid with a monopolar electrode until the pseudocapsule of the fibroid was identified. Using traction and contraction, the myoma is separated from the surrounding myometrium as described by Andrea Tinelli et al.^9^. The surgical site was sutured with absorbable barbed suture V-Loc™ 180 (Medtronic). Large myomas were placed inside a bag and extracted using the extracorporeal tissue extraction technique through the umbilicus.

### Statistical analysis

The sample size was calculated according to data published in the literature, considering a difference in hemoglobin levels between both groups of 0.7 g/ dl, with a statistical power of 80% and an alpha error of 5%.

Randomization of the sample was performed using EPIDAT 4.2 (Consellería de Sanidade, Xunta de Galicia, Spain; Organización Panamericana de la Salud (OPS-OMS); CES University, Colombia.)

The data collected from the medical records was tabulated in a main database with SPSS version 23.0 (IBM Corp. Released 2008. IBM SPSS Statistics for Windows, version 23.0 Armonk, NY: IBM Corp) that was also used for statistical analysis. Absolute and relative frequency distributions of qualitative variables were calculated. The differences in the values of the quantitative variables were analyzed by the appropriate test in each case (T Student and the corresponding non-parametric tests). Qualitative variables were compared with the Pearson chi-square test. In all analyses, the level of statistical significance considered was 95% (*p* < 0.05).

### Ethics approval

IRB approval was obtained prior to initiating the study. Written informed consent was obtained from all the patients prior to enrolling in the study.

## Results

Base line characteristics of patients included in the study are summarized in Table 1. All the women included except one were of childbearing age (age min: 21 years, max: 46 years), most patients had no previous pregnancies (93% Group A vs 83% Group B, *p* = 0.30). A 15.0% (n:12) of women were overweight (BMI > 25) and 4 (5.0%) were obese (BMI > 30), which was similar in both groups (Mean BMI Group A: 22.5 3.5 vs Group B: 23.4 4.0, *p* = 0.30).

**Table 1:** Patient characteristics.

		Group A (LM) n: 40	Group B (TOUA) n: 40	*p* value
Age (mean, IQR)		38 (35–41)	37 (35.4–39.0)	0.32
BMI (mean ± SD)		22.5 ± 3.5	23.4 ± 4.0	0.30
Parity (n, %)	0	37 (93%)	33 (83%)	0.30
	1	1 (3%)	5 (13%)	
	2	1 (3%)	1 (3%)	
	3	1 (3%)	0 (0%)	
	7	0 (0%)	1 (3%)	
Number of myomas (n, %)	1	13 (33%)	13 (33%)	0.77
	2	13 (33%)	9 (23%)	
	3	3 (8%)	7 (18%)	
	4	5 (13%)	5 (13%)	
	5	2 (5%)	2 (5%)	
	More than 5	4 (10%)	4 (10%)	
Diameter of largest myoma (mm) (mean ± SD)		74.9 ± 20.3	77.5 ± 24.7	0.61
Preoperative Hb (g/dl) (mean ± SD)		13.4 ± 1.2	13.3 ± 1.2	0.70

LM, laparoscopic myomectomy; TOUA, temporary occlusion of uterine arteries; Hb, Hemoglobin.

There were no significant differences in the number of myomas removed in both groups: most of the patients had 1 or 2 fibroids (66% Group A vs 56% Group B), 26% had between 3 to 5 fibroids, and a small percentage of patients had more than 5 fibroids (only 4 patients (10%) in each group). The largest diameter of the myomas was similar in both groups ranging from 2–15 cm.

The hemoglobin level prior to the intervention was similar in both groups (min:9.5 g/dl, max: 15.4 g/dl).

Perioperative data are summarized in Table 2. All surgeries were completely performed laparoscopic with no conversion to laparotomy needed.

**Table 2:** Operative and postoperative endpoints.

	Group A (LM) n: 40	Group B (TOUA) n: 40	*p* value
Operative time (min) (mean ± SD)	82.7 ± 25.0	92.1 ± 35.4	0.17
Blood loss (ml) (mean ± SD)	394.4 ± 231.0	227.1 ± 247.2	0.025
Postoperative Hb (g/dl) (mean ± SD)	11.1 ± 1.2	11.4 ± 1.4	0.43
Difference Hb pre-postop (mean ± SD)	2.3 ± 0.9	1.9 ± 1.1	0.15
Postoperative hospital stay (days) (mean, IQR)	1.0 (1.0–1.0)	1.0 (1.0–2.0)	0.17

LM, laparoscopic myomectomy; TOUA, temporary occlusion of uterine arteries.

The operative time was slightly longer in the intervention group (Group B), although the difference was not statistically significant (82.7 ± 25.0 min vs 92.1 ± 35.4 min, *p* = 0.17). Patients included in both groups were discharged 1–2 days after surgery.

Estimated blood loss was significantly lower in the clip placement group (Group B) (227.1 ± 247.2 ml vs 394.4 ± 231.0 ml, *p* = 0.025), although the postoperative hemoglobin levels or mean hemoglobin difference were not statistically significant (*p* = 0.43, *p* = 0.15 respectively). Two patients (one of each group) required postoperative blood transfusion, and one patient required hospital readmission due to an infected hematoma that was medically managed. No other complications were documented.

## Discussion

Lately, women frequently decide to delay their age of conception. As a result, more women present with symptomatic fibroids expressing the desire for future fertility. Laparoscopic myomectomy (LM) is a safe and effective technique for the treatment of women with fibroid uterus who wish to preserve future fertility. The laparoscopic approach has advantages over laparotomy of better and faster recovery, less postoperative pain, and lower risk of postoperative adhesion formation^10,11^.

However, it requires an experienced surgeon since it is frequently associated with a large amount of intraoperative blood loss, especially in cases of large myomas with an intramural component.

To prevent excessive intraoperative bleeding, different strategies have been proposed, including preoperative treatments with GnRH analogs, intramyometrial vasopressin injection, intraoperative misoprostol administration, intravenous oxytocin, and placement of a tourniquet around the cervix, among other options^2,11–13^.

The uterine artery is a branch of the internal iliac artery. It goes through the cardinal ligament and ascends through the broad ligament at both sides of the uterus, giving the arcuate arteries that penetrate the myometrium and endometrium, giving the radial arteries. In the presence of myomas, the arcuate arteries follow an irregular pattern until they reach the fibroids providing a dense vascular cortex^14^.

Laparoscopic uterine artery occlusion (LUAO) may be performed permanently by bipolar coagulation or suture ligation, or temporarily by placing titanium surgical clips. Liu et al.^6^ were the first to use surgical clips for LUAO using the lateral access, although clamping the uterine arteries approaching them with posterior or anterior access has also been described^2,15^. Titanium surgical clips were first used by Shao et al.^16^ in combination with vasopressin inyection to decrease uterine vascularization in surgical excision of caesarean scar ectopic pregnancies.

LUAO decreases the amount of bleeding by decreasing blood flow and pressure over the uterus, improving hemostasis and coagulation^15^ without causing myometrial ischemia. It is a quick procedure that does not increase the surgical time by more than 10–20 min in experienced hands^2^. The temporary lower amount of blood perfusion to the uterus as a result of LUAO reduces the need for bipolar coagulation, generating fewer scar tissue that could negatively impact future reproductive outcomes^17^, a larger amount of fibroids can be easily removed if present, it allows the surgeon to operate more comfortably and quickly with better visualization of the surgical field^17^. However, it requires to be performed by an experienced surgeon to prevent injuring nearby structures.

In our series, the estimated blood loss was lower in the patient of the LUAO group than in the LM group (227.1 ± 247.2 ml vs 394.4 ± 231.0, *p* = 0.025). Our difference (167.3 ml) is higher than the 103.7 ml reported by Sanders et al. in their meta-analysis after evaluating 1,569 patients with LUAO prior to laparoscopic or laparotomic myomectomy vs 1,302 patients with myomectomy without temporary artery occlusion^18^. They also found a small difference in the hemoglobin values of 0.6 g/dl that could be considered clinically similar to the one found by us in this study (0.4 g/dl), although in our case, the difference was not statistically significant probably due to the small sample size. In another meta-analysis, Tranoulis et al.^19^, analyzed 750 laparoscopic myomectomies without arterial occlusion compared to 873 performed with prior LUAO, showing that there was a reduction in the intraoperative estimated blood loss that ranged from 36.3 to 190 ml lower when LUAO was performed (109–270 ml). Peng et al.^17^ found a greater difference (178.0 ± 104.1 ml vs 258.1 ± 119.5, *p* < 0.05), and added as an advantage that having less bleeding allowed more myomas could be removed (4 vs 3, *p* < 0.05). Ji et al.^20^ also reported a lower estimated blood loss when performing LUAO (36.3 ± 8.7 ml) versus no intervention (126.7 ± 26.3 ml) before laparoscopic myomectomy. Jin et al.^21^ analyzed a group of patients undergoing LM alone, LM with LUAO with or without temporary bilateral ligation of utero-ovarian arteries, concluding that the lowest amount of intraoperative blood loss was achieved when temporary bilateral ligation of the utero-ovarian arteries was performed in addition to LUAO without increasing surgical time. Yang et al.^22^ conducted a multicenter study including 324 patients undergoing LUAO prior to LM and 180 women undergoing LM without artery obliteration, reporting an estimated blood loss of 83.6 ± 53.7 ml vs 109 ± 58.4 ml respectively. The temporary or permanent LUAO has similar impact on intraoperative blood loss^14^.

In our series, one patient from each group required to receive a blood transfusion (2.5%), which is lower than the rate previously described of 4 to 20%^1^ but similar to the frequency described by Yang et al.^22^ (1.85% in cases of LUAO and 6.11% in LM). The first case was a 43-year-old woman with a pre-operative hemoglobin of 9.5 g/dl who had a myoma of 13 cm. No LUAO was performed, and the estimated blood loss was 700 ml. The second case was a 29-year-old woman with a 9 cm myoma who underwent LUAO. Her starting hemoglobin was 13.5 g/dl, and the estimated blood loss was 1200 ml. Both received only 2 Units of packed red blood cells and recovered well after the blood transfusion.

Among other complications, one case had a postoperative infection. She was a thirty-eight-year-old woman who had three fibroids removed; the diameter of the largest fibroid was 10 cm. She required hospital readmission 4 days after surgery because of a fever without elevated white count due to a pelvic hematoma that spontaneously resolved after treatment with intravenous antibiotics.

Some authors recommend performing a laparotomy when the patient has fibroids larger than 8–10 cm or more than 3 fibroids, although the surgical approach depends largely on the surgeon’s experience^23^. In our series, 36 patients (45%) had fibroids with a size equal to or greater than 8 cm, 19 of which were randomly assigned to perform LUAO. We did not have the need to convert to laparotomy in any of the cases despite the large size of the fibroids. Other authors have also demonstrated the efficacy of LAUO in laparoscopic-assisted myomectomy^24^, a technique specially intended for the resection of large myomas.

It is important to note that once the excision of the fibroid is completed, the speed and the use of adequate surgical suturing technique is essential to reduce blood loss. In our case series, in both groups, we used the barbed suture to close the surgical site immediately after excision of the fibroids, which has the benefit of maintaining the tension of the thread without the need to tie knots^12^.

The operative time was longer in the LAUO group, however, it did not reach statistical significance (82.7 25.0 min vs 92.1 35.4 min, *p* = 0.17), similar as previously reported in the literature^18,19^. Ji et al.^20^ also found no difference in LAUO using clips requiring 55.1 ± 10.6 min for the resection of a single myoma or 120 ± 20.2 min when multiple myomas were resected.

It has been suggested that performing permanent LAUO decreases the chance of recurrence of fibroids^17–19^. LAUO reduces the blood supply to the myoma capsule, which becomes necrotic if not completely resected during the myomectomy procedure, while the rest of the myometrium would be revascularized through anastomosis from the utero-ovarian artery and ovarian pedicle. In addition, other smaller myomas not removed during the initial surgery may also become necrotic^17^. However, other studies in which LAUO is temporary have not shown such a decrease in recurrence^20,21^, perhaps because the temporary occlusion allows adequate reperfusion of the surgical site, and necrosis does not occur.

The impact of performing LAUO on future fertility is still unknown. There is a decrease in fertility in cases of uterine artery embolization previous to LM^25^, but the effect of a permanent LAUO on endometrial vascularization, embryo implantation, and early embryo development is unclear^14^. A temporary LAUO can produce transient hypoxia at the endometrial level, followed by revascularization from the rich network of collateral vasculature, ensuring revascularization of the uterus without impacting future fertility^26^. Sanders et al.^27^ recently conducted a meta-analysis looking at the impact of LUAO on the live birth rate. They included 689 women, of whom 470 had undergone permanent or transient LUAO by laparoscopy or laparotomy prior to myomectomy. No differences were found in the rate of live births (OR 0.89, *p* = 0.64), nor in pregnancy rate or ovarian reserve.

Our study is one of the very few randomized trials evaluating temporary occlusion of uterine arteries and utero-ovarian ligaments before laparoscopic resection of myomas. We acknowledge several limitations of our study, including the small sample size and the fact that the surgeries were performed by a single surgeon, which limits the external validity of our study.

Future studies should evaluate the influence of new treatments that may decrease the volume of the myoma prior to surgical removal. New generations of well-trained laparoscopic surgeons can be trained to apply these innovative techniques. It will also be interesting to evaluate the impact of LUAO when performing robotic-assisted surgery for the resection of myomas.

## Conclusion

Transient bilateral occlusion of the uterine and uteroovarian arteries prior to laparoscopic myomectomy is a safe procedure that reduces blood loss during the procedure without increasing operative time.

## Data Availability

The datasets used and/or analysed during the current study are available from the corresponding author on reasonable request.
